# Cloning and biochemical characterization of a novel lipolytic gene from activated sludge metagenome, and its gene product

**DOI:** 10.1186/1475-2859-9-83

**Published:** 2010-11-07

**Authors:** Li JunGang, Zhang KeGui, Han WenJun

**Affiliations:** 1 School of Life Sciences and Biotechnology, Mianyang Normal University. Mianyang, 621000, PR China; 2 Department of life science, Huainan Normal University. Huainan, Anhui 232001, PR China

## Abstract

In this study, a putative esterase, designated EstMY, was isolated from an activated sludge metagenomic library. The lipolytic gene was subcloned and expressed in *Escherichia coli *BL21 using the pET expression system. The gene *estMY *contained a 1,083 bp open reading frame (ORF) encoding a polypeptide of 360 amino acids with a molecular mass of 38 kDa. Sequence analysis indicated that it showed 71% and 52% amino acid identity to esterase/lipase from marine metagenome (ACL67845) and *Burkholderia ubonensis *Bu (ZP_02382719), respectively; and several conserved regions were identified, including the putative active site, GDSAG, a catalytic triad (Ser203, Asp301, and His327) and a HGGG conserved motif (starting from His133). The EstMY was determined to hydrolyse *p*-nitrophenyl (NP) esters of fatty acids with short chain lengths (≤C8). This EstMY exhibited the highest activity at 35°C and pH 8.5 respectively, by hydrolysis of *p*-NP caprylate. It also exhibited the same level of activity over wide temperature and pH spectra and in the presence of metal ions or detergents. The high level of stability of esterase EstMY with unique substrate specificities makes it highly valuable for downstream biotechnological applications.

## Introduction

Lipolytic enzymes are ubiquitous α/β hydrolyzing enzymes existing in animals, plants, and microbes. The enzymes contain esterases (EC3.1.1.1) and lipases (EC3.1.1.3) which catalyze the hydrolysis and synthesis of fatty acid esters including acylglycerides [1]. Due to some useful features such as broad substrate specificity, stability in organic solvents and regio-/enantioselectivity, lipolytic enzymes of microbial origin are widely used in industrial biotechnology, such as production of fine chemicals, pharmaceuticals, and fine chemicals synthesis [2-4].

Modern biotechnology has a steadily increasing demand for novel biocatalysts, thereby prompting the development of new experimental approaches to find and identify novel biocatalyst-encoding genes. Based on the direct cloning of the metagenome [5] for the construction of large clone libraries, metagenomics allows access to new sequences, genes, complete pathways and their products by multiple screening possibilities. With the advent of the metagenome approach, the so far uncultured microorganisms (estimated to more than 99%) [6-10] are now more readily accessible, resulting in an exponential increase in the number of potential biocatalysts. Indeed, the metagenomic approach was useful in mining novel lipolytic enzymes from environmental samples, and also, several genes encoding esterases have been isolated in metagenomic libraries prepared from highly diverse bacterial communities, including marine sediment [11-13], soils [8,10,14,15], drinking water biofilm [10], pond and lake water [16,17], and tidal flat sediment [18]. Some of these enzymes display enhanced characteristics, therefore, searching for novel lipolytic enzymes still attracts considerable attention.

Pre-studies based on 16S rDNA library have extensively expanded our knowledge of microbial diversity in activated sludge from sewage treat plant, including members of varied un-culturable groups (unpublished data). Here, we report the cloning, sequence analysis, and biochemical enzymatic characterization of a novel esterase, EstMY, from an activated sludge derived metagenomic library. Our report demonstrates that metagenomics is a powerful approach in mining new industrial enzymes. The esterase EstMY constituted a new member of family IV of bacterial lipolytic enzymes.

## Materials and methods

### Sampling

Activated sludge was collected from a sewage treatment plant treating nitrogen-containing aromatic wastewater on September 2008 in Mianyang City, SiChuan Province.

### Bacterial strains, plasmids, and culture

The starting strains and plasmids used in this study are listed in Table 1. *E. coli *was grown at 37°C in Luria-Bertani (LB) medium supplemented with appropriate antibiotics [19]. When required, ampicillin was added at a final concentration of 100 μg/ml, kanamycin at 25 μg/ml, and chloramphenicol, at 12.5 μg/ml.

**Table 1:** Starting bacterial strains and plasmids used in this study

Strain or plasmid	Description	Source or reference
**Strains**		
*E. coli *TOP10	*lac*х*74 recA1 deoR F - mcrA *∆ (*mrr-hsdRMS-mcrBC*) *ϕ80 lacZ*∆*M15*∆ *araD139*∆ *(ara-leu)7697 galU galK*	Transgen
*E. coli *EPI300™-T1R	*[F- e14-(McrA-) D(mcrC-mrr) (TetR) hsdR514 supE44 supF58 lacY1 or D(lacIZY)6 galK2 galT22 metB1 trpR55 l-]*	Epicentre
*E. coli *BL21(DE3)	*F-, ompT, hsdSB (rB-, mB-), dcm, gal, λ(DE3), pLysS, Cmr*	Novogen
*E. coli *EPI300-FosD11L2	Positive clone from Fosmid genomic library, which carries the lipolytic gene	This study
*E. coli *TOP10-EstMY	Positive clone from sublibrary, which carries the *EstMY *gene fragment	This study
*E. coli *BL21(DE3)-EstMY	Positive clone, which carries the pEstMY-His expression vector	This study
**Plasmids**		
pCC1FOS	Cloning vector; Chl^r^	Epicentre
pUC18	Cloning vector; Ap^r^	Takara
pET28a	Expression vector; Km^r^	Novagen
FosD11L2	pCC1FOS, which carries the *estMY *gene cluster (31 kb)	This study
pUC18-EstMY	pUC18, which carries the complete lipolytic gene (*estMY*)	This study
pEstMY-His	pET28a carrying amplified *Hin*dIII -*Nde*I fragment containing lipolytic gene (e*stMY*)	This study

Ap^r^, ampicillin resistant; Chl^r^, chloramphenicol resistant; Km^r^, kanamycin resistant.

### DNA preparation and manipulation

*E. coli *cells were transformed by the calcium chloride procedure [19]. Recombinant plasmid DNA was isolated by the method of Birnboim and Doly [20]. For sequencing, this DNA was further purified by polyethylene glycol precipitation [19]. Restriction enzymes, T4 DNA ligase and calf intestinal alkaline phosphatases were purchased from New England Biolabs (Ipswich, USA) or Takara (Tokyo, Japan) and used according to the manufacturers' instructions. BugBuster Ni-NTA His. Bind Purification Kit was purchased from Novagen (Code No. NV70751-3, Novagen).

### Construction of metagenomic DNA library and related sublibrary

Activated sludge DNA extraction was carried out as previously described using SDS and proteinase K treatment [21], and removing humic acids (HAs) prior to DNA extraction was conducted by removing HAs buffer, 100 mmol/L Tris-HCl pH 10.0, 100 mmol/L Na_4_P_2_O_7 _100 mM, Na_2_EDTA, 1.0% PVP, 100 mM NaCl, 0.05% Triton X-100 [22]. Approximately 150 μg of metagenomic DNA was run on a preparative pulsed-field gel (Bio-Rad CHEF DR^®^III; 0.1-40 s switch time, 6 V/cm, 0.5× TBE buffer, 120° included angle, 16 h) and the appropriate size of DNA ranging from 30-45 kb was isolated, electroeluted and dialyzed against 0.5× TE buffer for further Fosmid library construction. The purified DNA fragments were end-repaired by End-repaired enzyme mix. After drop dialysis and concentration, the blunt-ended, 5'-phosphorylated DNA was ligated into the cloning-ready Copycontrol pCC1FOS vector, and the recombinant molecules were packaged into ྔ phage followed by phage transfection to *E. coli *EPI300 by using protocols described in MaxPlax™ Lambda packaging kit (Epicentre Biotechnologies, Madison, Wisconsin, USA). A fosmid clone showing strong lipolytic enzyme activity on a tributyrin agar plate was selected for further characterization and designated FosD11L2. The DNA was purified from the selected clone, partially digested with *Sau*3AI in order to obtain 3-5 kb DNA fragments, ligated to the pUC18 vector and transformed into *E. coli *TOP10 cells (Transgen). Transformants were selected on LB (ampicillin, 100 μg/ml) plates containing 1% (v/v) tributyrin as the indicator substrate [23].

### Genetic characterization and sequence analysis

The lipolytic DNA fragment obtained from positive clone *E.coli *TOP10-EstMY was sequenced with primer walking method by SinoGenoMax Co. Ltd (Chinese National Human Genome Center, Beijing). The ORFs were analyzed using DNASTAR (Lynnon Biosoft) software and ORF finder online analysis http://www.ncbi.nlm.nih.gov/projects/gorf/, Database searches for protein sequences was performed using BLAST and FASTA programs [24,25]. Peptide sequences of various enzymes or subunits were extracted from National Center for Biotechnology Information (Washington, D.C).

### Phylogenetic analysis

Deduced amino acid sequences of 12 lipolytic enzymes were subjected to protein phylogenetic analysis. A phylogenetic tree was generated using the neighbor joining method of Saitou and Nei [26] with MEGA 4.0 software [27]. A total of 6 sequences were aligned with the CLUSTAL_W program [28] and visually examined with BoxShade Server program. The length of each branch pair represents the evolutionary distance between the sequences.

### Heterologous expression of gene *estMY *and purification of recombinant EstMY

To express EstMY, the full length of the *estMY *gene was amplified by PCR with a pair of primers *estMY*-f and *estMY*-r (Table 2), in which the high fidelity PrimeSTAR™HS DNA Polymerase (code: DR010SA, Takara) was used. The integrity of the nucleotide sequence of all newly constructed plasmids was confirmed by DNA sequencing. The primer pairs with restriction enzyme sites (underlined) for *Hin*dIII and *Nde*I were designed to generate an N-terminal His-tag of the recombinant esterase. The *estMY *gene was cloned into an expression vector, pET28a (+) and the recombinant plasmid p*estMY*-His was transformed into *E. coli *BL21 (DE3) cells. When the cell density at 600 nm reached around 0.6, expression of recombinant EstMY protein was initiated by addition of 0.6 mM isopropylthio-β-D-galactoside and continued cultivation for additional 4 h. Cells were harvested by centrifugation at 5,000 ×g for 5 min, washed twice with ice-cold 50 mM sodium phosphate buffer (pH 8.0) and resuspended in the same buffer containing 10 mM imidazole, disrupted by sonification in an ice-water bath (60 times, 5s). Recombinant EstMY esterase was applied to metal-chelating chromatography using Ni-NTA affinity chromatography (Novagen) according to the manufacturer's instructions.

**Table 2:** Primers used in the study

Primer	Sequence 5'-3'	Description
HTFP061	GTACAACGACACCTAGAC	Sequencing primer for pCC1FOS™
HTRP062	CAGGAAACAGCCTAGGAA	Sequencing primer for pCC1FOS™
M13 primer RV	CAGGAAACAGCTATGAC	Sequencing primer for pUC18
M13 primer M2	AGCTGTTCACCGAAGTGCTG	Sequencing primer for pUC18
*EstMY *-W1F	CGCCCCTTTCGACCAGCAACG	Genomic walking primer for *estMY *gene
*EstMY *-W2F	CTACGCCGACCTCACCGGCCT	Genomic walking primer for *estMY *gene
*EstMY *-W1R	GAGGGGTGTGCGGGGATGCG	Genomic walking primer for *estMY *gene
*EstMY *-W2R	GACGTAGCCGCCGCCGTGAAG	Genomic walking primer for *estMY *gene
*EstMY *-F	GGCAT**ATG**GCCGCGCCCGTTCCGCCCATCAG *Nde*I	Forward primer for *estMY *gene
*EstMY *-R	GGAAGCTTCTACGCTGCCGCCCTAGCGCCGATG*Hin*dIII	Reverse primer for *estMY *gene

The *Nde*I and *Hin*dIII sites are underlined. The start codon is in bold.

Polyacrylamide gel electrophoresis of enzyme in the presence of sodium dodecyl sulfate (SDS) was carried out by the method of Sambrook and Russell [19].

### Characterization of recombinant EstMY and biochemical properties

The purified EstMY was subjected to a series of biochemical analysis, including determing the pH optimum, temperature optimum, substrate specificity, and effects of various detergents and metal ions. All measurements were carried out in triplicate. The values were the mean of the data. The substrate specificity of the purified EstMY protein was performed using the following substrates of *p*-NP-fatty acyl esters [23,29]: acetate (C2), butyrate (C4), hexanoate (C6), caprylate (C8), decanonate (C10), laurate (C12), myristate (C14) and palmitate (C16). The enzyme was incubated with the ester derivatives (0.5 mM) in 5 ml Tris-HCl buffer (50 mM, pH 8.0) at 30°C for 10 min. The reaction was quenched by adding 5 ml trichloroacetic acid (0.5 mM) and then recovered the original pH value with 5.15 ml NaOH (0.5 mM). The enzymatic activity was measured by monitoring the *p*-nitrophenoxide production by absorbance at 405 nm against an enzyme-free blank, which was measured using a Ultraspec 3000 UV/vis spectrometer (Amersham Biosciences, Sweden) [30,31]. One unit of enzyme activity was defined as the amount of activity required to release 1 μmol *p*-NP per minute under the above condition. The highest activities of enzyme assay using the substrate (i. e. *p*-NP-caprylate) was defined as the 100%. To determine the presence of esterase activity, the triglyceride derivative 1,2-di-*O*-lauryl-*rac*-glycero-3-glutaric acid 6'-methylresorufin ester (DGGR) (Sigma Aldrich) was used as a chromogenic substrate, and the formation of methylresorufin was analyzed spectrophotometrically at 580 nm [32-34]. *Candida rugosa *lipase (Sigma Aldrich) was used as a positive control.

The optimum temperature of purified EstMY was determined by assaying lipolytic enzyme activities in a 50 mM Tris-HCl buffer (pH 8.0) for a temperature range of 20-65°C, in which *p*-NP-caprylate (0.5 mM) acted as substrate. Optimal pH was determined by examining the activity of the enzyme after incubation at 35°C for 10 min using *p*-NP-caprylate (0.5 mM) as substrate. The buffers used were: 50 mM phosphate buffer (pH 5.0-7.5), 50 mM Tris-HCl (pH 8.0-10.5).

Various metal ions (CoCl_2_, CaCl_2_, ZnCl_2_, MgCl_2_, K_2_SO_4_, FeSO_4_, CuCl_2_, Ni(NO_3_)_2_, and FeCl_3_), and chelating agent EDTA at final concentrations of 5 mM were added to the enzyme in 50 mM Tris-HCl (pH 8.0), whereafter it was assayed for esterase activity following preincubation at 35°C. Effect of detergents or reductors on esterase activity was determined by incubating the enzyme for 30 min at 35°C in 50 mM Tris-HCl (pH 8.0), containing Triton X-100, Tween 20, Tween 80, β-mercaptoethanol, 1,4-dithiothreitol (DTT), sodium dodecyl sulfate (SDS), cetyltrimethyl ammonium bromide (CTAB), phenylmethanesulfonyl fluoride (PMSF), diethylpyrocarbonate (DEPC). The concentrations of metal ions, EDTA, detergents, and surfactants used were 5 mM, 3 mM, and 0.5% (v/v), respectively. The activity of the enzyme preparation in the absence of metal ions and detergents before incubation was defined as the 100% level.

### Nucleotide sequence accession number

The DNA sequence of EstMY from activated sludge was deposited in GenBank under accession number of HM366454.

## Results and discussion

### Construction and screening of a metagenomic library

One hundred micrograms of prokaryotic DNA was extracted per gram of wet-weight activated sludge, and 1.5 μg of size-selected, pulsed field gel-purified high-molecular-weight (HMW) DNA suitable for fosmid library construction was obtained. Three hundred nanograms of 30-45 kb purified metagenomic DNA was ligated into the copy control pCC1FOS vector and then tranfected into *E. coli *EPI300-T1^R^, producing a metagenome library of more than 7,0000 fosmids with insert size ranging from 27 kb to 38 kb, with an average size of 32 kb, covering approximately 2.1 Gbp of the total metagenomic DNA. Given an average prokaryotic genome of approximately 5 Mbp, the metagenome library theoretically reached the size of over 400 prokaryotic genomes. The prokaryotic origin of the library was confirmed by end-sequencing of randomly selected fosmids and comparison with known ORFs in NCBI. Expression screening of the fosmid library for hydrolytic activity based on the hydrolysis of emulsified tributyrin (1%) resulted in the finding of a recombinant clone, FosD11L2, forming a clear zone on the indicator plate. In order to identify the hydrolytic gene within a fragment of 31 kb, the insert was subject to further subcloning.

### Subcloning and identification of the esterase gene

**Figure 1:**
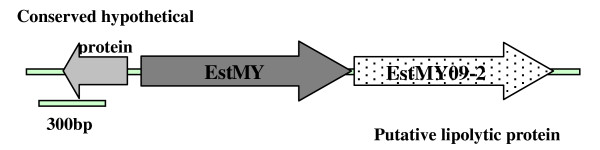
**Sequencing of subclones (FosD11L2) expressing lipolytic activity resulted in the assembly of a 2,609 bp contig**. Three major ORFs with conserved domains were identified: *estMY*, encoding a novel esterase EstMY; a putative conserved hypothetical protein, with homology to a cytidylate kinase; and an ORF (EstMY09-2) encoding a putative lipolytic protein.

The DNA insert (31 kb) of fosmid D11L2 was partial digested by *Sau*3AI and subcloned into prepared pUC18 vector, producing a subclone library of more than 3,000 clones with an average insert size of 3.5 kb. One hundred and fifty subclones were screened for lipolytic activity. Among the 9 positive sub-clones forming a clear zone on the indicator plates, one sub-clone that expressed extracellular lipase/esterase activity was sequenced from both ends and the sequences were assembled into a contig of 2,680 bp. An ORF of 1,083 bp encoding a putative lipase/esterase (named EstMY) of 360 amino acids was identified. A second ORF encoding a putative lipolytic enzyme, designated EstMY-092, was identified as well as an additional putative ORF encoding a conserved hypothetical protein (Figure 1).

Amino acid sequence alignment indicated that this EstMY exhibited low identity with other esterase/lipases. EstMY shared the highest (71%) sequence identity with the ACL67845 esterase/lipase isolated from a marine metagenome library, 65% sequence identity to Est25 screened from a soil metagenomic library [35], followed by the putative lipase/esterase from other environmental samples (50-65% identity), the putative alpha/beta hydrolase from *Burkholderia ubonensis *Bu and *Parvibaculum lavamentivorans *DS-1 (ZP_02382719, 52% identity; and YP_001412150, 49% identity, respectively), members of the family IV hydrolases.

**Figure 2:**
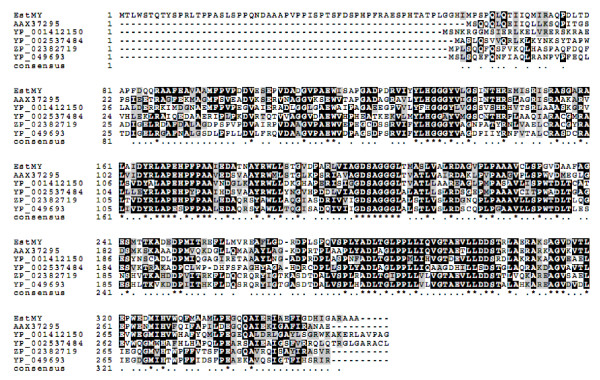
**Conserved sequence blocks from multiple sequence alignment of EstMY from activated sludge metagenomic library and other related proteins**. Sequences alignment was carried out with CLUSTALW [28] and BoxShade Server http://www.ch.embnet.org/software/BOX_form.html. AAX37295, lipase/esterase from marine metagenome; YP_001412150, alpha/beta hydrolase domain-containing protein from *Parvibaculum lavamentivorans *DS-1; YP_002537484, alpha/beta hydrolase domain-containing protein from *Methylobacterium populi *BJ001; ZP_02382719, putative acetyl-hydrolase from *Burkholderia ubonensis *Bu; YP_049693, putative acetyl-hydrolase from *Pectobacterium atrosepticum *SCRI1043.

**Figure 3:**
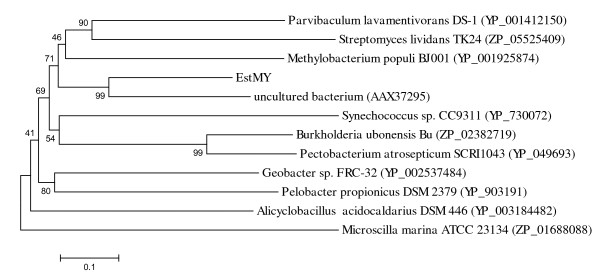
**Phylogenetic analysis of EstMY and closely related proteins**. Phylogenetic analysis was performed using the program MEGA4.0. Except for EstMY, the protein sequences for bacterial lipolytic enzymes were retrieved from GenBank http://www.ncbi.nlm.nih.gov. The numbers at node indicate the bootstrap percentages of 1000 resamples.

Various lipases and esterases contain the conserved active site motif of the pentapeptide GXSXG with a serine acting as the catalytic nucleophile, a conserved aspartate or glutamate and a histidine, together constituting a catalytic triad [2], organized in the α/β hydrolase fold [36]. The amino acid sequence alignment to bacterial lipolytic enzymes retrieved from GenBank http://www.ncbi.nlm.nih.gov, identified the conserved motifs, including the putative active site GDSAG (Figure 2). Thus, EstMY probably uses a catalytic triad consisting of the serine (Ser203) in the GDSAG active site, the aspartate (Asp301) and the highly conserved histidine (His327) for catalysis. Moreover, EstMY contains a HGGG conserved blocks (starting from His133), which corresponds to a family IV characteristic motif (HGG), which is in close proximity to the active site contributing to the formation of the oxyanion hole that is likely to participate directly in the catalytic process [2,11,37]. Furthermore, to clarify the phylogenetic relationship of the EstMY with other esterases or lipases, a neighbour joining phylogenetic tree was constructed using the amino acid sequence of the lipolytic enzymes. As shown in Figure 3. In this tree, EstMY formed a distinct group with the uncultured bacterium protein (AAX37295), which is located closest to the branch of putative acetyl-hydrolase (accession number ZP_02382719) of strain *Burkholderia ubonensis *Bu, esterase (accession number ZP_05525409) from *Streptomyces lividans *TK24, and also, alpha/beta hydrolase domain-containing protein (accession number YP_001412150 and YP_001925874 respectively) from *Parvibaculum lavamentivorans *DS-1 and *Methylobacterium populi *BJ001 respectively, which constitute family IV lipases. These results suggest that the EstMY is a new member of family IV lipases.

### Expression and purification of recombinant EstMY

**Figure 4:**
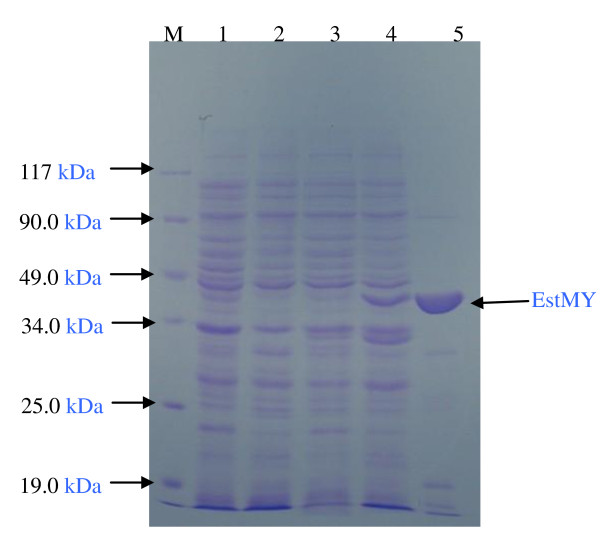
**SDS-polyacrylamide gel of overexpressed esterase EstMY in *E. coli***. Lane 1: molecular weight protein marker (Tiangen, Cat. No: MP203); lane 2, *E. coli*/pET28a: total protein extract, as negative control; lane 3: induced culture of *E. coli*/pET28a, as negative control; lane 4: total protein extract *E. coli*/pEstMY-His; lane 5, total protein extract, induced culture of *E. coli*/pEstMY-His; lane 6: purified EstMY (38 kDa).

To investigate the property of this EstMY, *estMY *gene was expressed as an N-terminal His-tag fusion protein using pET-28a(+) expression system in *E. coli *BL21(DE3). The recombinant protein was analyzed by SDS-PAGE and Coomassie brilliant blue staining (Figure 4). These results indicate that recombinant EstMY protein is expressed (Mw, about 38 kDa), as which correlated well to the predicted full length of EstMY. The purity of the purified protein was more than 98% according to SDS-PAGE analysis.

### Substrate specificity of EstMY

**Figure 5:**
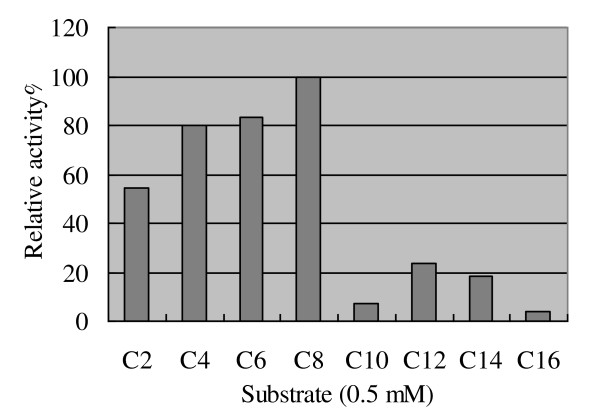
**Substrate specificity of overexpressed and purified esterase EstMY**. Specific activity of hydrolysis of different *p*-nitrophenyl esters. *p*-NP acetate (C2), *p*-NP butyrate (C4), *p*-NP hexanoate (C6), *p*-NP caprylate (C8), *p*-NP decanoate (C10), *p*-NP laurate (C12), *p*-NP myristate (C14), and *p*-NP palmitate (C16). Relative activity was shown as the percentage of the activity of the activity towards 4-nitrophenyl caprylate. All measurements were performed in triplicate.

We expressed EstMY as a hexahistidine-tagged (His-tagged) protein and investigated its chain length substrate specificity using *p*-nitrophenyl esters (Sigma). Results showed EstMY was able to hydrolyse *p*-nitrophenyl esters with acyl chains up to 14 carbons (*p*-nitrophenyl myristate), with the highest activity towards short-chain fatty acids (C2, C4, C6 and C8), while much lower towards long-chain fatty acids (>C8) (Figure 5). Moreover, the EstMY was not able to hydrolyse the triglyceride derivative 1, 2-di-*O*-lauryl-*rac*-glycero-3-glutaric acid 6'-methylresorufin ester (DGGR) (data not shown), while DGGR was able to form chromogenic product methylresorufin by the true lipase from *Candida rugosa *(positive control). Furthermore, EstMY showed no fluorescence on olive oil plates with rhodamine B, which indicated that EstMY is a true esterase [29,32,38,39].

### Effect of temperature and pH on EstMY

**Figure 6:**
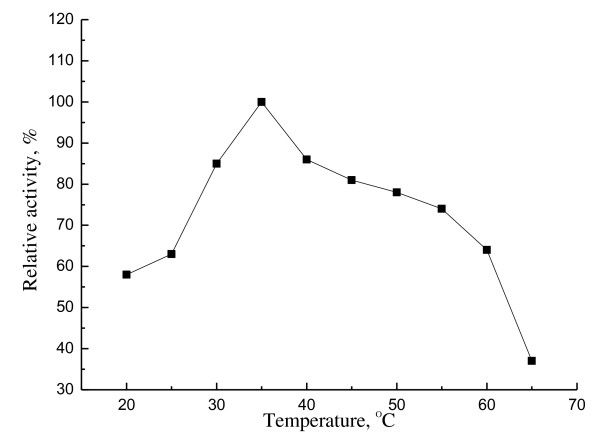
**Apparent temperature optimum of esterase EstMY**. Relative activity of *p*-NP-caprylate hydrolysis at different temperatures by purified EstMY. The activity was determined at different temperatures at pH 8.0 in 50 mM Tris-HCl buffer. The activity at 35°C was set as 100% (3,936 U/ml). All measurements were performed in triplicate.

**Figure 7:**
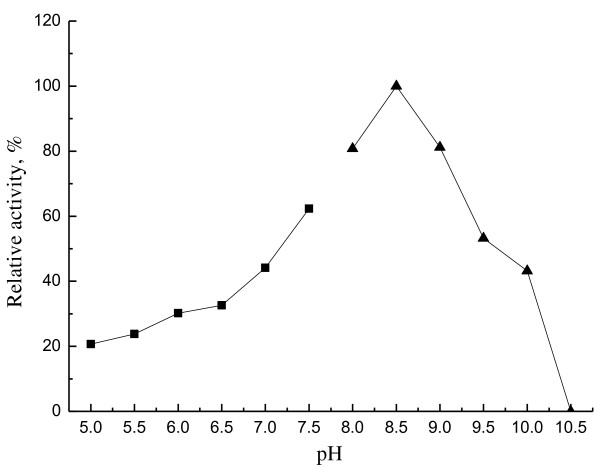
**Effect of pH on the purified esterae EstMY**. Relative activity of *p*-NP-caprylate hydrolysis was performed in various pH buffers at 35°C (pH 5.0-7.5, 50 mM phosphate buffer; pH 8.0-10.5, 50 mM Tris-HCl buffer). The activity at pH 8.5 was set as 100% (4,615 U/ml). All measurements were performed in triplicate.

Esterase activity of EstMY was determined from 20°C to 65°C. The purified EstMY showed highest activity at 35°C. It showed a broader temperature spectrum and retained over 37% activity at 65°C (Figure 6). However, h1Lip1 from marine sediment metagenome showed a bad thermostability because there was no activity left after incubation at 40°C for 30 minutes [29]. And also, the esterase showed activity in a rather broader pH range of 7.0-10.0. Maximal activity was observed at pH 8.5 and lost activity at pH 10.5 (Figure 7).

### Effect of metal ions on esterase

The effects of metal ions and ethylenediamine tetraacetic acid (EDTA) on the EstMY esterase activity were investigated by measuring the residual enzyme activity in their presence and depicted in Table 3. Among metal ions tested, the esterase activity was slightly increased by Co^2+ ^(126%), Ca^2+ ^(104%) and K^+ ^(103%). Furthermore, the esterase activity was inhibited by Ni^2+^, Zn^2+^, and Mg^2+^, moreover, almost totally inhibited by Cu^2+ ^, and Fe^3+ ^(7% and 10% residual acitivity respectively), while the chelating agent EDTA had no effect, suggesting this esterase is not a metalloenzyme.

**Table 3:** Effect of metal ions on esterase activity

Compounds	Concentration (mM)	Relative activity (%)
Control	0	100.0 ± 2.9
CoCl_2_	5	126.4 ± 2.1
K_2_SO_4_	5	103.2 ± 3.6
FeSO_4_	5	100.9 ± 2.6
CuCl_2_	5	7.8 ± 2.7
Ni(NO_3_)_2_	5	36.2 ± 4.3
EDTA	5	102.7 ± 3.2
FeCl_3_	5	10.9 ± 3.4
CaCl_2_	5	104.1 ± 3.7
ZnCl_2_	5	23.7 ± 1.8
MgCl_2_	5	79.7 ± 2.6

Activity without metal ions was set as 100% (4,897 U/ml). All measurements were repeated three times.

### Effect of detergents and reductors on esterase

The effects of detergents and reductors on esterase activity are shown in Table 4. A significant increase in lipolytic activity was observed with addition of 3 mM CTAB (130%), 0.5% Triton X-100 (129%), Tween 80 (138%), and Tween 20 (156%), after 0.5 h preincubation with detergents at 35°C. Moreover, 3 mM β-mercaptoethanol and DTT did not affect the lipolytic activity (101% and 106%, respectively), whereas DEPC and SDS had a strong inhibitory effect on esterase activity. In accordance to our results, Nawani et al. [40] also found a total inactivation of activity in the presence of SDS but an enhanced activity in the presence of Triton X-100, Tween 80, and Tween 20. Interestingly, the esterase EstMY activity was not impacted by 3 mM PMSF, suggesting EstMY may possess a lid structure, which could eliminate the inhibition effect of PMSF. This is a special characteristic of carboxylesterases [11,41,42] and site-directed mutagenesis of amino acid Ser203 will be carried out to confirm the function of Ser203.

**Table 4:** Effect of detergents and enzyme inhibitors on esterase activity

Compounds	Concentration	Relative activity (%)
Control	0	100.0 ± 2.1
β-mercaptoethanol	3 mM	101.7 ± 2.6
DTT	3 mM	106.9 ± 4.9
CTAB	3 mM	129.7 ± 2.2
DEPC	3 mM	38.6 ± 2.7
PMSF	3 mM	101.3 ± 4.1
SDS	3 mM	12.3 ± 2.9
Triton X-100	0.5%	129.6 ± 4.6
Tween 80	0.5%	138.4 ± 2.1
Tween 20	0.5%	156.7 ± 3.3

Activity without detergents and enzyme inhibitors was set as 100% (4,970 U/ml). All measurements were repeated three times.

In conclusion, we identified a new esterase EstMY belonging to family IV lipases, whose encoding gene was isolated from activated sludge of a sewage treatment plant treating nitrogen-containing aromatic wastewater. EstMY is expected to show high potential for downstream biotechnological applications including synthetic organic chemistry. This was confirmed by its extensive biochemical characterization, which revealed the enzymes substrate specificity, wide pH and temperature spectra, and also, stability towards addictives including metal ions and detergents. Future work will establish the structure of this enzyme to gain more information about its catalytic mechanism. Our research also demonstrated the potential of metagenome strategy in bioprospecting novel genes and biocatalysts and expanded our knowledge of biocatalyst diversity, especially for bacterial esterases. Enlargement of the lipases/esterases pool can be an immediate source of genetic modification, or yield enzymes that can be further specialized by directed evolution, and also, this would optimize their industrial applications.

## Competing interests

The authors declare that they have no competing interests.

## Authors' contributions

JGL participated in the design of experiments, and carried out the study and drafted the manuscript. KGZ carried out the SDS-PAGE experiment, sequence alignment and enzyme characteristics analysis. WJH conceived the study, and participated in its design and coordination and helped to draft the manuscript. All authors read and approved the final manuscript.
